# Telmisartan reverses antiretroviral-induced adipocyte toxicity and insulin resistance *in vitro*

**DOI:** 10.1177/1479164118757924

**Published:** 2018-02-21

**Authors:** Sudeep P Pushpakom, Antonysunil Adaikalakoteswari, Andrew Owen, David J Back, Gyanendra Tripathi, Sudhesh Kumar, Philip McTernan, Munir Pirmohamed

**Affiliations:** 1 Department of Molecular and Clinical Pharmacology, The Wolfson Centre for Personalised Medicine, University of Liverpool, Liverpool, UK; 2 Warwick Medical School, University of Warwick, Coventry, UK; 3 Department of Biomedical Sciences, University of Westminster, London, UK

**Keywords:** HIV, antiretroviral, insulin resistance, metabolic disease, telmisartan, adipocyte

## Abstract

**Background:**

Antiretroviral therapy in HIV-positive patients leads to insulin resistance which is central to the pathogenesis of various metabolic abnormalities and cardiovascular disease seen in this patient group. We have investigated the dose–response relationship of telmisartan, an antihypertensive, on adipocytes *in vitro* in order to determine whether it may have metabolic beneficial effects.

**Methods:**

Using *in vitro* chronic toxicity models (3T3-F442A murine and primary human adipocytes), we evaluated the effects of different concentrations of telmisartan on adipocyte differentiation and adipogenic gene expression using lipid accumulation assays and real-time polymerase chain reaction, respectively. Adipokine secretion and expression of insulin signalling mediators were evaluated using enzyme-linked immunosorbent assays.

**Results:**

Telmisartan partially reversed the deleterious effects of antiretrovirals on adipocyte lipid accumulation, expression of adipogenic regulators (peroxisome proliferator receptor-gamma and lipin 1), adipokine secretion and expression of the insulin signalling mediator pAkt_Ser473_. The metabolic effects of telmisartan followed a non-monotonic response with the maximal effect observed at 5 µM in the primary human adipocyte model.

**Conclusion:**

Telmisartan has beneficial metabolic effects in adipocytes *in vitro*, but its potential to reduce antiretroviral-induced cardiometabolic disease in HIV-infected individuals needs to be evaluated in a well-designed adequately powered clinical trial.

## Introduction

Combination antiretroviral therapy (cART) is the mainstay of treatment in HIV. It has improved the morbidity and mortality associated with HIV, turning it into a chronic disease. However, cART, together with the virus itself, increases the risk of various metabolic complications, including obesity,^1^ type 2 diabetes mellitus (T2DM) and cardiovascular disease (CVD).^2^ Indeed, CVD is the leading cause of death in HIV-infected patients on cART with a linear increase in the incidence of myocardial infarction observed with long-term cART exposure.^3^

Insulin resistance (IR) is central to the development of cardiometabolic disease,^4^ being present in 21% of HIV patients on antiretroviral (ARV) therapy.^5^ *In vitro* as well as single-drug studies in both healthy^6^ and HIV-infected patients^7^ have shown that IR can be induced by both protease inhibitors (PIs) and nucleoside reverse transcriptase inhibitors (NRTIs). Although newer ARVs are increasingly used in clinical practice, IR still remains an important problem; HIV patients (n = 328) randomised to tenofovir disoproxil fumarate/lamivudine (TDF/3TC) with either boosted atazanavir (ATV) or boosted darunavir or raltegravir showed a 1.9-fold increase in homeostatic model assessment–IR (HOMA-IR) within 4 weeks.^8^ Importantly, HIV-associated metabolic disease results in increased healthcare burden; a recent study in the United States identified the management of IR/diabetes to be the biggest contributor to the cost burden and resource use among all HIV-related adverse events studied.^9^

Adipose tissue is an important determinant of IR and may therefore play a key role in cART-associated metabolic disease. Adipose tissue has also been shown to be a reservoir for HIV and a source of chronic inflammation.^10^ Clinical interventions to arrest or reverse cART-associated adipose-mediated IR are a potential strategy to reduce the incidence of T2DM and CVD in HIV-positive patients. To this end, insulin sensitisers such as thiazolidinediones and metformin have been trialled, but results from randomised clinical trials in HIV-positive patients have been disappointing and sometimes deleterious.^11–13^ There is therefore a need for novel clinical interventions that can reduce cART-induced IR in HIV-positive individuals.

Preliminary *in vitro* studies have suggested that telmisartan (TEL), an angiotensin II receptor blocker (ARB), reduces cART-induced adipose dysfunction by inhibition of the renin–angiotensin system (RAS).^14^ In addition to being an ARB, TEL is also a partial agonist at the peroxisome proliferator receptor-gamma (PPARγ) receptor,^15^ a key regulator of adipose tissue metabolism.^16^ In this article, we further evaluate the effect of TEL on cART-induced adipocyte dysfunction and IR in a novel chronic *in vitro* toxicity model, in addition to assessing its concentration–response relationship.

## Materials and methods

### Materials

Murine 3T3-F442A cells were a kind gift from Prof Karen Chapman (University of Edinburgh). Primary human abdominal subcutaneous preadipocytes were obtained commercially from age- and sex-matched healthy donors (n = 3; body mass index < 25 kg/m^2^; Promocell, Heidelberg, Germany). Collection of adipose tissue was approved by local ethics committee and all donors gave informed consent. None of the donors had any known medical conditions (i.e. hypertension, CVD, thyroid disorders, renal disorders, diabetes or chronic pain conditions) or were on endocrine, anti-inflammatory, statin, thiazolidinedione or antihypertensive therapy. Lopinavir (LPV), ritonavir (RTV), ATV and rosiglitazone (ROSI) were purchased from Santa Cruz Biotechnology (Dallas, TX, USA) and TEL was provided by Boehringer Ingelheim GmbH (Ingelheim, Germany). Adipocyte media were obtained from PromoCell. TaqMan gene expression assays [PPARγ and lipin 1 (LPIN1)] and TaqMan Gene Expression Master Mix were purchased from Life Technologies Ltd (Paisley, UK). Singleplex and multiplex enzyme-linked immunosorbent assays (ELISAs) for adipokines [adiponectin, interleukin-6 (IL-6), tumour necrosis factor-α (TNF-α) and resistin] were obtained from Merck Millipore (Hertfordshire, UK) and Life Technologies Ltd. A colorimetric assay for free fatty acid release was obtained from Abcam (Cambridge, UK). Estimation of phospho-Akt (pAkt_Ser473_) and total Akt was performed by sandwich ELISA kits obtained from Thermo Fisher Scientific (Paisley, UK).

### Methods

#### In vitro chronic adipocyte toxicity model: ARVs accumulate extensively within the adipocytes,^10^ and thus, we used a chronic in vitro toxicity model to mimic this

Briefly, both 3T3-F442A murine cells and primary human subcutaneous adipocytes were cultured, induced to differentiate as described previously,^17^ and treated with PIs with or without TEL and/or ROSI throughout adipocyte differentiation. For 3T3-F442A, the cells were cultured with Dulbecco’s Modified Eagle’s medium (Sigma-Aldrich, Dorset, UK) and 10% foetal calf serum followed by the initiation of differentiation using 10 mg/mL insulin (Sigma-Aldrich). Primary human preadipocytes were cultured in a Preadipocyte Growth Medium which is a low-serum (5% v/v) medium optimised for the expansion of human preadipocytes. Once the cells became 70%–80% confluent, differentiation was induced by culturing them in the Preadipocyte Differentiation Medium, a serum-free medium, for 3 days followed by further maintenance of differentiating adipocytes in the Adipocyte Nutrition Medium. Drug treatment was started 48 h post initiation of differentiation and carried out every 48 h over a period of 10 days (or 12 days in the case of primary human adipocytes). The effects of PIs were tested over a wide concentration range (1–20 µM) including their near-Cmax values (RTV and LPV: 10 µM; ATV: 4.4 µM). We initially selected two different concentrations of TEL (1 and 5 μM) based on the previous literature;^14,18^ but for further dose characterisation of TEL, we tested a range of concentrations (0.5–20 μM). TEL was coincubated with each of the PIs and added at the same time. ROSI (10 μM), a PPARγ agonist, was coincubated with LPV only in the primary human adipocyte model as a comparator.

#### Measurement of cell viability

Viability of differentiating 3T3-F442A and primary human adipocytes was assessed using the 3-(4,5-dimethylthiazol-2-yl)-2,5-diphenyltetrazolium bromide (MTT) assay. MTT measures mitochondrial metabolism as a surrogate marker of cell viability.^19^ The cells were incubated with the ARVs at serial concentrations (0.01–100 µM) for 4 days. On day 4, the cells were incubated with MTT and the absorbance of the resultant formazan product was measured at 560 nm.

#### Lipid accumulation assay

Lipid accumulation in differentiating adipocytes was assessed on day 10 (3T3-F442A cells) or day 12 (primary human adipocytes) of differentiation using Oil Red O (Sigma, Dorset, UK) as previously described.^14^ Lipid-bound dye was extracted using 70% isopropyl alcohol and staining was quantified at 520 nm. The drug-treated cells were compared against the vehicle-treated control (methanol).

#### RNA extraction and gene expression

Total RNA was isolated using the RNeasy kit (Qiagen, Manchester, UK). Total RNA was reverse transcribed using the TaqMan^®^ reverse transcription kit (Life Technologies Ltd). Gene expression of *PPARγ* and *LPIN1* were assessed by real-time polymerase chain reaction (PCR) using TaqMan Assays-on-Demand gene expression assays on a 7900HT Fast Real-Time PCR System (Life Technologies Ltd).

#### Assessment of lipolysis

Free fatty acid concentration in the conditioned media of primary human adipocytes was determined using the Free Fatty Acid Quantification Assay Kit as per manufacturer’s instructions (Abcam). Briefly, palmitic acid standard (1 nmol/μL) was used to prepare the standard curve dilution; the reaction plates were prepared and incubated in the dark at room temperature for 30 min and the absorbance was measured using a microplate spectrophotometer (Beckman Coulter DTX880 Multimode Detector) at 595 nm.

#### Estimation of adipokines

Adipokine (adiponectin, IL-6, resistin and TNF-α) concentrations in the conditioned media were determined on day 10 (3T3-F442A) or day 12 (primary human adipocytes) post differentiation using bead-based Milliplex Mouse Sandwich Multiplex ELISA kits (Merck Millipore) and Human Singleplex ELISA kits (Life Technologies Ltd), respectively. The ELISA kits used had the following detection limits: adiponectin, 5.2 pg/mL (murine) and 100 pg/mL (human); IL-6, 5.3 pg/mL (murine) and < 1 pg/mL (human); TNF-α, 11.2 pg/mL (murine) and < 2 pg/mL (human) and resistin, 6.1 pg/mL (murine) and 10 pg/mL (human).

#### Estimation of phosphorylated Akt content

Phosphorylated Akt (serine residue 473) as well as total Akt was quantitated by a sandwich ELISA, as recommended by the manufacturer (Thermo Fisher Scientific). Briefly, the diluted lysates were applied to 96-well plates containing immobilised monoclonal antibodies specific for human Akt and incubated for 2 h at room temperature. A pS473 Akt standard and the samples were pipetted into the wells, followed by washing and incubation with a rabbit antibody (Cell Signalling, MA, USA) specific for AKT phosphorylated at serine 473. Following washing, a horseradish peroxidase-labelled anti-rabbit IgG was added and washed; a substrate solution [3,3′,5,5′-tetramethylbenzidine (TMB)] was added to produce colour and the absorbance was read at 450 nm.

### Statistical analysis

Data are presented as mean ± standard deviation (SD) for at least three independent experiments to ensure reproducibility. Statistical significance was determined using the non-parametric Mann–Whitney U test (IBM SPSS Statistics, version 22). The threshold of significance was set at *p* < 0.05.

## Results

### ARVs cause adipocyte cytotoxicity

**Figure 1.**
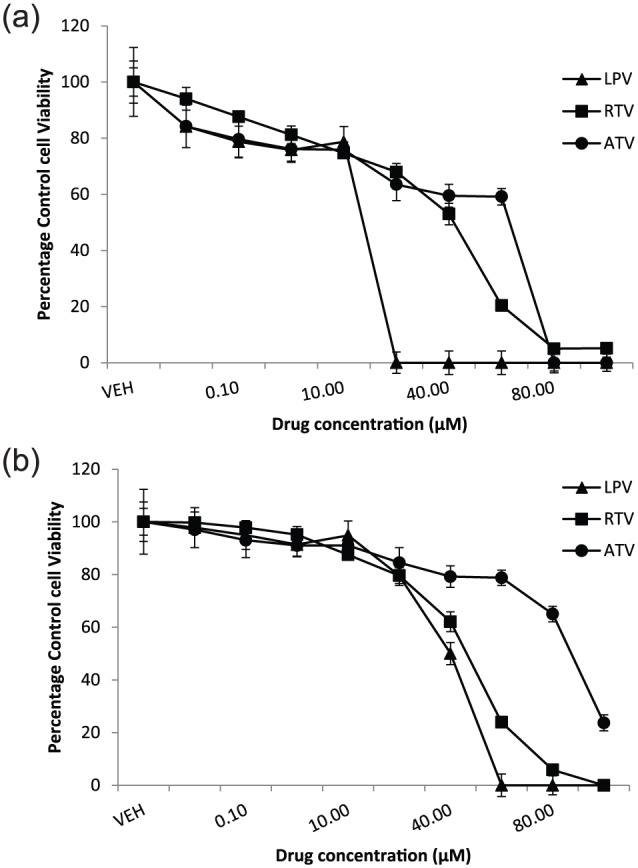
Cytotoxicity profile of protease inhibitors in differentiating (a) 3T3-F442A and (b) primary human adipocytes. Data are expressed as mean (n = 3) ± SD. RTV: ritonavir; LPV: lopinavir; ATV: atazanavir.

Cytotoxicity was observed with all ARVs during differentiation of preadipocytes and followed a similar trend in both 3T3-F442A and primary human cells. In differentiating 3T3-F442A adipocytes, the rank order for cytotoxicity was LPV (IC_50_ = 14 µM) > RTV (IC_50_ = 48 µM) > ATV (IC_50_ = 66 µM; Figure 1(a)). In primary human adipocytes undergoing differentiation, the rank order for cytotoxicity was LPV (IC_50_ = 28 µM) > RTV (IC_50_ = 38 µM) > ATV (IC_50_ = 84 µM; Figure 1(b)).

### TEL reverses ARV-induced inhibition in adipocyte lipid accumulation

**Figure 2.**
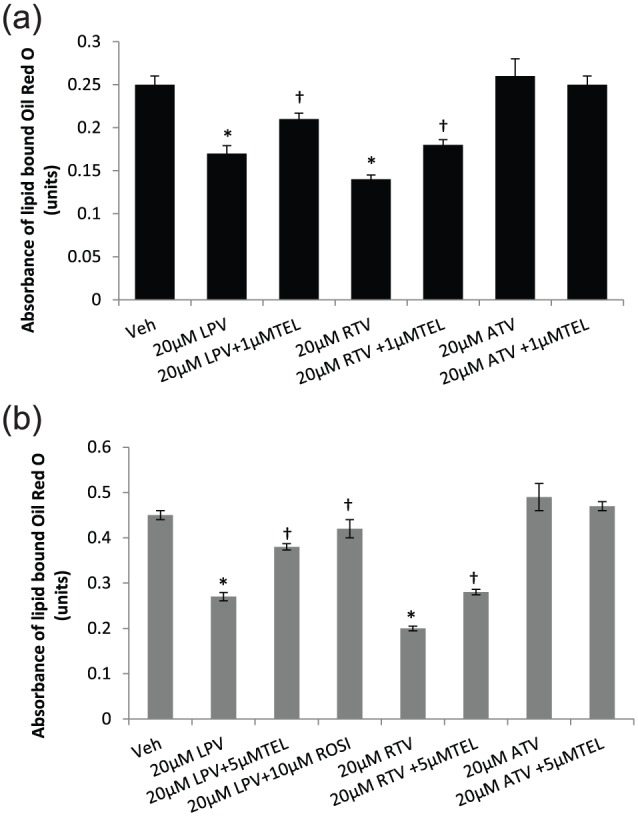
Lipid accumulation in differentiating (a) 3T3-F442A and (b) primary human adipocytes following incubation with PIs with/without TEL or ROSI. ROSI was coincubated with LPV in the primary human adipocyte model only. Data are expressed as mean (n = 3) ± SD. (^*^p < 0.01, drug vs vehicle; ^†^*p* < 0.01, drug vs drug + TEL or drug + ROSI.). Veh: vehicle; RTV: ritonavir; LPV: lopinavir; ATV: atazanavir; TEL: telmisartan; ROSI: rosiglitazone.

In 3T3-F442A adipocytes, a dose-dependent reduction in lipid accumulation was observed for LPV (at 20 µM, a reduction of 32% in mean absorbance, *p* < 0.01) and RTV (at 20 µM, 44% reduction, *p* < 0.01) but not for ATV [at 20 µM, 4% increase, non-significant (NS)] in comparison with the vehicle-treated controls (Figure 2(a); full concentration response data are given in Supplementary Information); 1 µM TEL partially reversed (*p* = 0.01) the RTV- and LPV-induced reduction in lipid accumulation in 3T3-F442A adipocytes (Figure 2(a)).

In primary human adipocytes, LPV and RTV (40% and 55% decrease, respectively, in comparison with the vehicle-treated control, *p* < 0.01) but not ATV (9% increase; NS) inhibited lipid accumulation (Figure 2(b)). Both TEL (5 µM) and ROSI (10 µM) reversed (*p* < 0.01) the ARV-induced inhibition of lipid accumulation partially in primary human adipocytes (Figure 2(b)). The effect shown by ROSI was stronger than that by TEL.

### TEL reverses ARV-induced downregulation of PPARγ and LPIN1 gene expression

#### PPARγ

**Figure 3.**
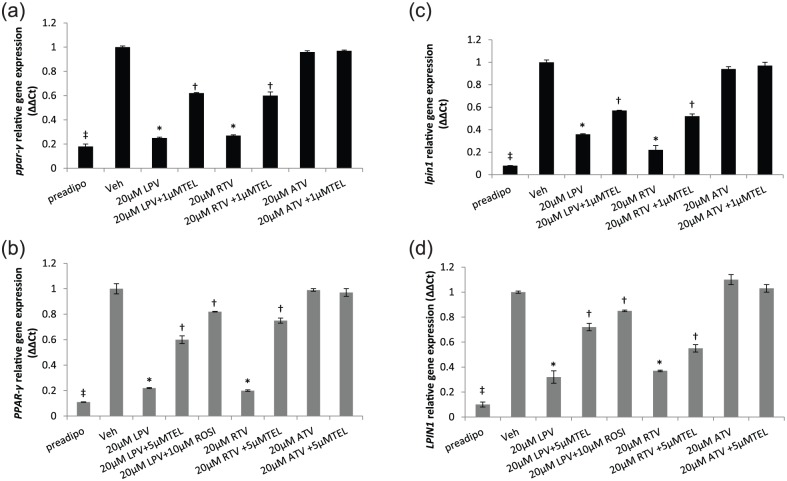
Expression of PPARγ: (a) 3T3-F442A and (b) primary human adipocytes; expression of LPIN1: (c) 3T3-F442A and (d) primary human adipocytes, following incubation with PIs with/without TEL or ROSI. ROSI was coincubated with LPV in the primary human adipocyte model only. Data are expressed as mean (n = 3) ± SD. (^*^p < 0.01, drug vs vehicle; ^†^*p* < 0.01, drug vs drug + TEL or drug + ROSI; ^‡^*p* < 0.01, preadipocyte vs vehicle.). Preadipo: preadipocyte; Veh: vehicle; RTV: ritonavir; LPV: lopinavir; ATV: atazanavir; TEL: telmisartan; ROSI: rosiglitazone.

In 3T3-F442A adipocytes, both LPV and RTV (75% and 73% downregulation, respectively, *p* < 0.01), but not ATV, downregulated *PPARγ* gene expression in comparison with the vehicle-treated control. This was partially but significantly reversed by 1 µM TEL (Figure 3(a)). A similar result was observed in primary human adipocytes (LPV and RTV with 78% and 80% downregulation, respectively) which was partially reversed by 5 µM TEL (LPV + TEL, *p* = 0.03; RTV + TEL, *p* = 0.01; Figure 3(b)).

#### LPIN1

Both LPV and RTV downregulated *LPIN1* gene expression in both 3T3-F442A adipocytes (LPV, 64%; RTV, 78%, *p* < 0.01; Figure 3(c)) and primary human adipocytes (LPV, 68%; RTV, 63%, *p* < 0.01; Figure 3(d)). In both models, this was partially reversed by 1 (3T3-F442A) or 5 µM (primary human adipocytes) TEL (Figure 3(c) and (d)). ATV did not have any effect on LPIN1 expression.

### TEL reverses ARV-induced changes in adipokine secretion

#### Adiponectin

**Figure 4.**
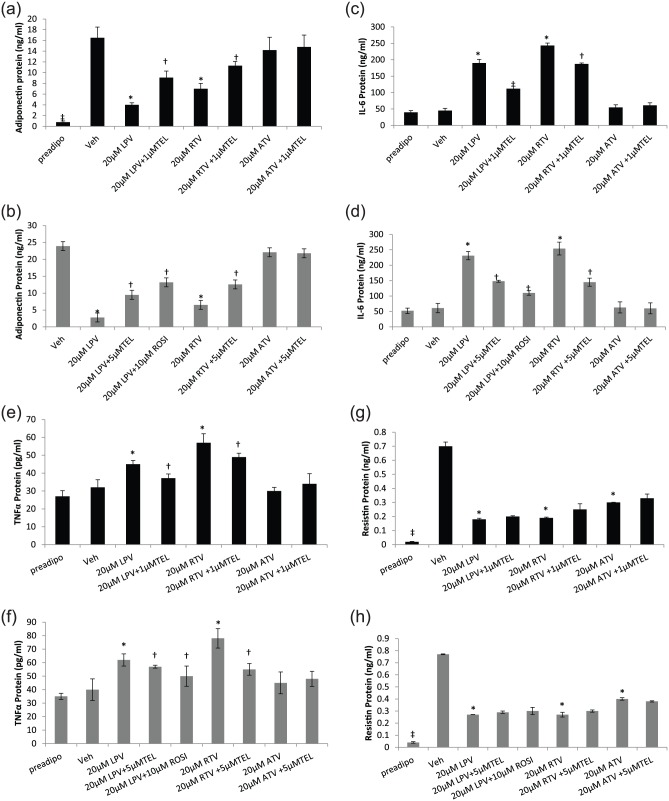
Adipokine secretion in differentiating 3T3-F442A and primary human adipocytes following incubation with PIs with or without TEL or ROSI: adiponectin: (a) 3T3-F442A and (b) primary human adipocytes; IL-6: (c) 3T3-F442A and (d) primary human adipocytes and TNF-α: (e) 3T3-F442A and (f) primary human adipocytes; resistin: (g) 3T3-F442A and (h) primary human adipocytes. ROSI was coincubated with LPV in the primary human adipocyte model only. Data are expressed as mean (n = 3) ± SD. (*p < 0.01, drug vs vehicle; ^†^*p* < 0.01, drug vs drug + TEL or drug + ROSI; ^‡^*p* < 0.01, preadipocyte vs vehicle.). Preadipo: preadipocyte; Veh: vehicle; RTV: ritonavir; LPV: lopinavir; ATV: atazanavir; TEL: telmisartan; ROSI: rosiglitazone.

In 3T3-F442A adipocytes, both LPV (4.0 ng/mL ± 0.4; *p* = 0.002) and RTV (7.0 ± 1.0; *p* = 0.001) but not ATV (14.2 ± 2.4) caused downregulation in secreted adiponectin protein in comparison to the vehicle-treated control (16.5 ± 2.0; Figure 4(a)). 1 µM TEL resulted in a significant but partial reversal of PI-induced downregulation of adiponectin (Figure 4(a)). A similar result was observed in primary human adipocytes (LPV, 88% reduction, *p* = 0.01; RTV, 73% reduction, *p* = 0.01; ATV, 7.5% reduction, NS); both TEL (5 µM) and ROSI (10 µM) were able to significantly reverse PI-induced downregulation of adiponectin (Figure 4(b)).

#### IL-6

Both LPV and RTV but not ATV increased the secretion of IL-6 in 3T3-F442A adipocytes (LPV, 190 ng/mL ± 11.3; RTV, 243 ± 7.9; both *p* < 0.01; ATV: 55 ± 8.0, NS) in comparison to the vehicle-treated control (45 ± 7.1; Figure 4(c)). A similar effect was also observed in primary human adipocytes for these PIs (LPV: 278% increase; RTV: 316% increase; both *p* < 0.01; Figure 4(d)). In both *in vitro* models, coincubation with TEL partially reversed PI-induced upregulation of secreted IL-6 (Figure 4(c) and (d)).

#### TNF-α

*LPV* (3T3-F442A, 45 pg/mL ± 2.1; primary human adipocytes, 62 pg/mL ± 4.2, both *p* < 0.001) and RTV (3T3-F442A, 57 ± 5.0; primary human adipocytes, 78 ± 5.7, both *p* < 0.01) but not ATV (3T3-F442A, 30 ± 2.0; primary human adipocytes, 45 ± 5.0) upregulated secreted TNF-α in comparison to the vehicle-treated control (3T3-F442A, 32 ± 4.3, and primary human adipocytes, 40 ± 5.0; Figure 4(e) and (f)). Coincubation with either 1 (3T3-F442A) or 5 µM TEL (primary human adipocytes) or 10 µM ROSI (primary human adipocytes only) significantly reversed PI-induced upregulation of TNF-α.

#### Resistin

All three PIs downregulated resistin in both murine (LPV: 74% decrease; RTV: 73% decrease; ATV: 57% decrease; all in comparison to vehicle-treated control; *p* < 0.01; Figure 4(g)) and primary human adipocytes (LPV and RTV: 65% decrease; ATV: 48% decrease; all in comparison to vehicle-treated control; *p* < 0.01; Figure 4(h)). Both TEL and ROSI (in primary human adipocytes only) showed a trend to reverse the PI-induced downregulation of resistin, but this was not significant in either of these models (Figure 4(g) and (h)).

### TEL reverses ARV-induced adipocyte lipolysis and inhibition of Akt phosphorylation in primary human adipocytes

**Figure 5.**
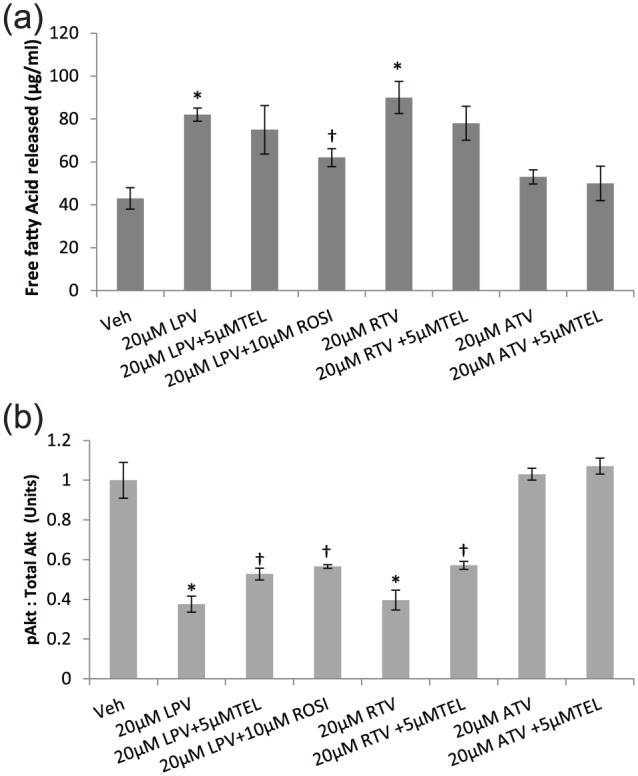
(a) Lipolysis and (b) expression of pAkt_Ser473_ in human primary adipocytes following incubation with PIs with or without TEL or ROSI. Data are expressed as mean (n = 3) ± SD. pAkt expression was adjusted to total Akt and data are expressed as mean ratio of absorbance. (^*^p < 0.01, drug vs vehicle; ^†^*p* < 0.01, drug vs drug + TEL or drug + ROSI.). Veh: vehicle; RTV: ritonavir; LPV: lopinavir; ATV: atazanavir; TEL: telmisartan; ROSI: rosiglitazone.

Both LPV (90% increase; *p* < 0.03) and RTV (109% increase; *p* < 0.01) but not ATV (23%; *p* = NS) resulted in an increase in free fatty acid levels in the conditioned media in primary human adipocytes suggesting enhanced lipolysis by these drugs (Figure 5(a)). Coincubation with 5 µM TEL reduced PI-induced lipolysis although the effect was statistically NS. However, 10 µM ROSI showed a significant partial reversal of ARV-induced lipolysis.

A significant reduction in the expression of pAkt_Ser473_ was observed with LPV (at 20 µM, 63% reduction, *p* < 0.01) and RTV (at 20 µM, 61% reduction; *p* < 0.01), but not with ATV, in comparison with the vehicle-treated controls (Figure 5(b)). Both LPV and RTV reduced pAkt_Ser473_ expression in a dose-dependent manner (see Supplementary Information). Coincubation with 5 µM TEL or 10 µM ROSI significantly reversed PI-induced downregulation of pAkt_Ser473_ (Figure 5(b)).

### Characterisation of optimal TEL dose to elicit metabolic effect

**Figure 6.**
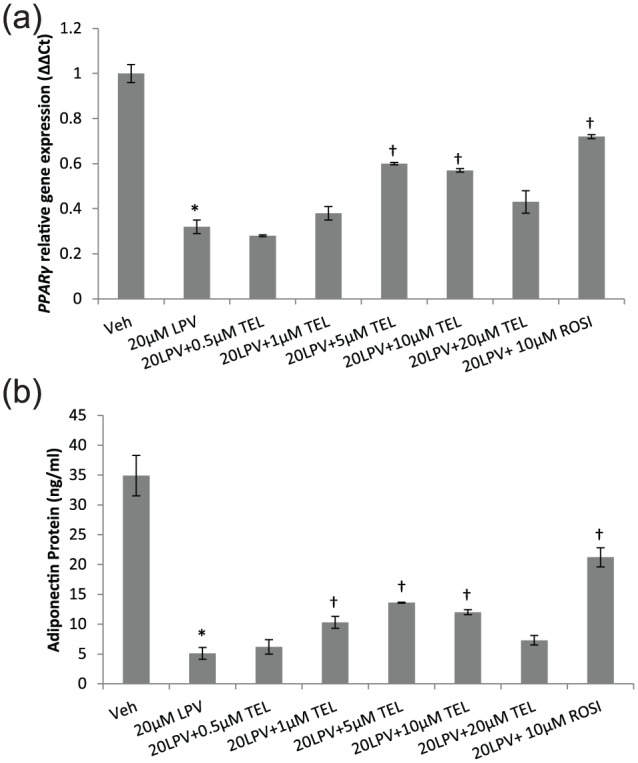
Dose–response relationship between TEL and *in vitro* metabolic effects: effect of TEL on (a) PPARγ and (b) secreted adiponectin over the full concentration range. Data are expressed as mean (n = 3) ± SD. (^*^p < 0.01, drug vs vehicle; ^†^*p* < 0.01, drug vs drug + TEL or drug + ROSI.). Veh: vehicle; LPV: lopinavir; TEL: telmisartan; ROSI: rosiglitazone.

Using secreted adiponectin and *PPARγ* gene as the exemplar markers, we evaluated the concentration–response relationship of TEL in the presence of LPV (20 µM) in primary human adipocytes. TEL significantly reversed LPV-induced inhibition of adiponectin at 1, 5 and 10 µM concentrations; for PPARγ, the effect of TEL was observed at 5 and 10 µM only. Importantly, in both instances, the maximal response for TEL was observed at 5 µM with TEL showing a non-monotonic dose response (Figure 6(a) and (b)).

## Discussion

We have confirmed the previously reported toxic effect of PIs on adipocytes and also identified how PIs affect novel adipokines such as LPIN1 and resistin. We also found that TEL results in a partial but significant reversal of ARV-induced adipocyte toxicity and, for the first time, characterised the concentration of TEL that elicits the maximal metabolic effect *in vitro*. We used 3T3-F442A cells as they are one of the most widely used *in vitro* models to study adipogenesis and are committed to differentiating into adipocytes.^20^ They have also been shown to develop a homogeneous population of mature adipocytes that are morphologically and biochemically similar to adipocytes *in situ*.^20^ The chronic drug treatment design enabled repeated drug exposure to the adipocytes over the entire period of adipocyte differentiation; this mimicked the *in vivo* situation where the long-term ARV drug treatment may result in cumulative adipocyte toxicity.^21^ LPV and RTV were toxic to adipocytes in both *in vitro* models, while ATV, a more lipid-friendly PI,^22^ was not, suggesting that the accumulation of certain ARVs over time may reduce cell viability in differentiating adipocyte populations in vivo and potentially deleteriously affect the fat cell turnover and thereby adipose tissue distribution.

Both LPV and RTV decreased lipid accumulation and messenger RNA (mRNA) expression of the adipogenic markers, PPARγ and LPIN1, consistent with their anti-adipogenic effects. By contrast, ATV did not show any effect on any of the above markers of lipid metabolism even at a concentration of 20 µM (>4 times its Cmax value of 4.4 µM). The contrasting effects of ATV support clinical data,^22^ which show that ATV has very little effect on body fat distribution in HIV patients. This is the first study to report an effect of PIs on *LPIN1*, a gene that encodes a magnesium-ion-dependent phosphatidic acid phosphohydrolase enzyme involved in triglyceride synthesis^23^ and a key factor in the maturation and maintenance of adipocyte differentiation.^24^ *LPIN1* is also a key transcriptional regulator of PPARγ and various genes involved in lipid metabolism.^25^ Interestingly, *LPIN1* mutations cause different types of severe human lipodystrophy syndromes^26^ although our previous work has failed to identify any association between *LPIN1* single-nucleotide polymorphisms and HIV lipodystrophy (HIVLD).^27^ The inhibitory effect of lipotoxic PIs on *LPIN1* could potentially be one of the mechanisms involved in the transcriptional downregulation of PPARγ caused by these drugs.

LPV and RTV had a profound effect on the secretory characteristics of the adipocyte regardless of the model used. Our results on adiponectin, IL-6 and TNF-α further highlight how certain PIs may interact with the adipokine network and regulate their transcription leading to adipocyte dysfunction and interference with insulin signalling. In addition, LPV and RTV but not ATV showed a significant reduction in the expression of phosphorylated form of Akt (Ser473); Akt is a serine/threonine kinase and a downstream target of phosphoinositide 3-kinase (PI3K) signalling, and phosphorylation of its serine residue at position 473 is an important step in the insulin signalling pathway.^28^

This study also explored the effects of PIs on resistin, an adipocyte-secreted protein which is implicated in IR. While higher circulating levels of resistin have been implicated in the development of IR^29^ and diabetes,^30^ its role in HIVLD is inconclusive. Some cross-sectional studies have reported an increase in circulating resistin levels in HIVLD patients,^31^ while other studies failed to find an association^32^ or even reported a reduction in resistin levels.^33^ We observed a significant reduction in the amount of resistin secreted by the adipocyte with all PIs including ATV although the ATV effect was comparatively less than those of other PIs. If resistin was involved in IR or adipocyte dysfunction, we would have expected its level to increase; our results suggest that secreted resistin might not be contributing directly to IR or adipocyte dysfunction in this ARV-treated cellular model. Previous clinical studies have reported a decrease in plasma resistin with TEL in diabetes patients;^34^ however, neither TEL nor ROSI had any effect on resistin in these *in vitro* models. It should be noted that in humans, resistin is primarily produced by cell populations other than adipocytes,^35^ including peripheral blood mononuclear cells, macrophages and bone marrow cells. This could potentially explain the discrepancy in resistin levels between clinical and *in vitro* studies.

TEL is widely used as an antihypertensive because of its ability to antagonise the effect of angiotensin II. However, TEL is highly lipophilic, and it has been suggested that its off-target effect on PPARγ could be beneficial in the treatment of metabolic disease and CVD.^36–38^ In this study, TEL was able to partially reverse the PI-induced inhibition in adipogenesis (lipid accumulation, expression of PPARγ and LPIN1), improve PI-induced reduction in adiponectin and expression of pAkt_Ser473_ (effect on insulin sensitivity), and reverse PI-induced upregulation in the secretion of proinflammatory markers, IL-6 and TNF-α. A previous *in vitro* study had shown that TEL improves adipocyte function following incubation with ARVs through blockade of the adipose RAS.^14^ It should be noted that PPARγ is also a modulator of adipocyte RAS and activation of PPARγ using full/partial agonists like ROSI or TEL could potentially counter the effects of RAS. By testing a wide range of concentrations of TEL (0.5–20 μM) on two exemplar markers, *PPARγ* gene and adiponectin protein, we observed TEL to show a non-monotonic response with the maximal effect observed at 5 μM in the primary human adipocyte model. This dose response shown by TEL here is different to that seen on blood pressure, which is linear and mediated by the angiotensin receptor, AT1R. It might very well be that both RAS and PPARγ play an independent role in the development of PI-induced adipocyte dysfunction; given that PPARγ full agonists such as ROSI suffer from serious adverse effects, ARBs such as TEL with dual activity on both PPARγ and RAS may offer an opportunity to reduce PI-induced toxicity.

## Limitations of the study

This study has not investigated the effect of ARVs (with/without TEL) in mature adipocytes; of course, the adipocyte population *in vivo* is a mixture of differentiating and differentiated adipocytes, but we felt it was important to focus on differentiating adipocytes, as harmful effects here would ultimately affect the population of differentiated adipocytes. This study did not assess the effect of PI drug combinations as used in the clinic; relating the concentration–response relationships *in vitro* to the in vivo situation is challenging because of differences that can occur in protein binding and drug distribution. It should be noted that we have only used three replicates (biological replicates) for each experiment in this study, but there was a high degree of reproducibility within the experiments. Taken together, our findings support the beneficial metabolic effects observed with TEL and open up the intriguing possibility that TEL could be used to prevent the increase in IR that is seen in HIV-infected individuals treated with ARVs.

## Conclusion

This study has shown that TEL has beneficial metabolic effects on adipocytes when given in combination with PIs and therefore has the potential to reverse adipocyte toxicity and IR mediated by PIs. The study also, for the first time, has characterised the dose response of TEL in human primary adipocytes. These *in vitro* findings now need to be validated in a clinical study which preferably not only evaluates, in a randomised fashion, the ability of TEL to reduce IR in vivo, but also identifies the optimal dose. This is currently being pursued in a phase IIb adaptive design clinical trial.^39^

## Supplementary Material

Supplementary material
