# Supplementary material for: Telmisartan reverses antiretroviral-induced adipocyte toxicity and insulin resistance in vitro
Source: Diab Vasc Dis Res. 2018 Feb 21;15(3):233–42. doi: 10.1177/1479164118757924 (PMC5949706; doi:10.1177/1479164118757924)
Supplement: Supplementary material [file Supplementary-Information_DVR757924.pdf]

Supplementary information:

A) 3T3-F442A adipocytes

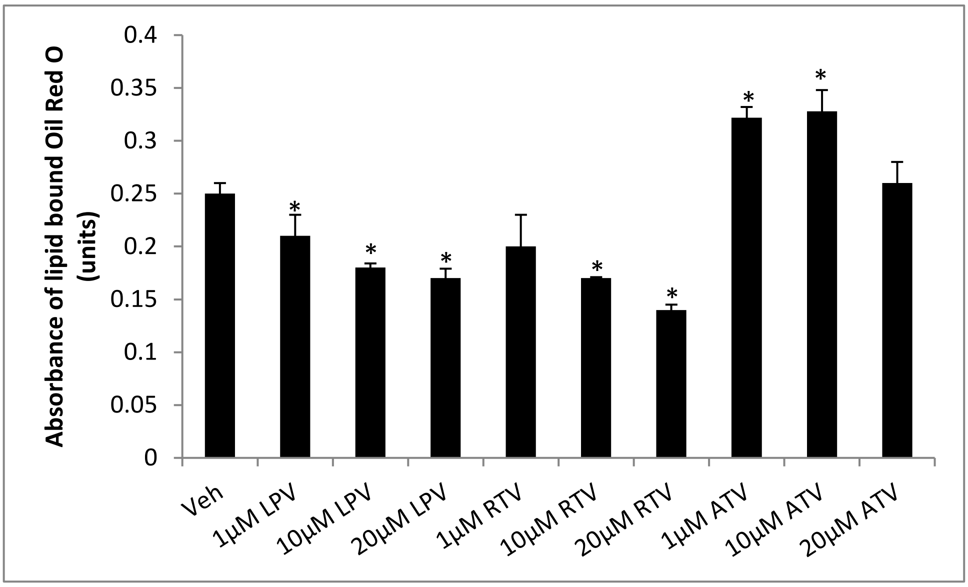

B) Primary human adipocytes

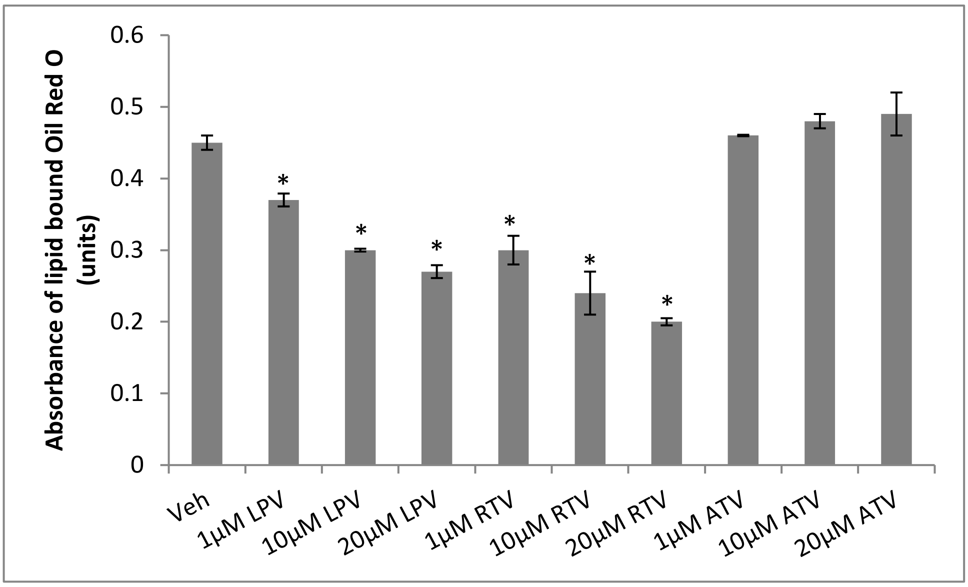

Figure 1. Lipid accumulation in differentiating 3T3-F442A (A) and human primary adipocytes (B) following incubation with serial concentrations of protease inhibitors. Data expressed as mean (n=3) ± SD. *=P<0.01; Drug v Vehicle.

A)

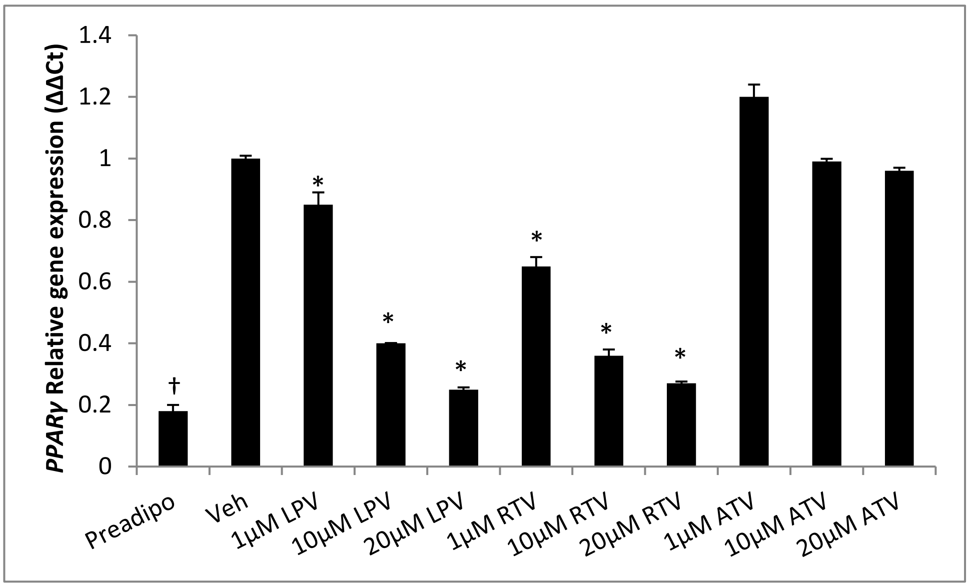
PPARγ Relative gene expression (ΔΔCt)
1.4
1.2
1
0.8
0.6
0.4
0.2
0
†
*
*
*
*
*
*
Preadipo
Veh
1µM LPV
10µM LPV
20µM LPV
1µM RTV
10µM RTV
20µM RTV
1µM ATV
10µM ATV
20µM ATV

B)

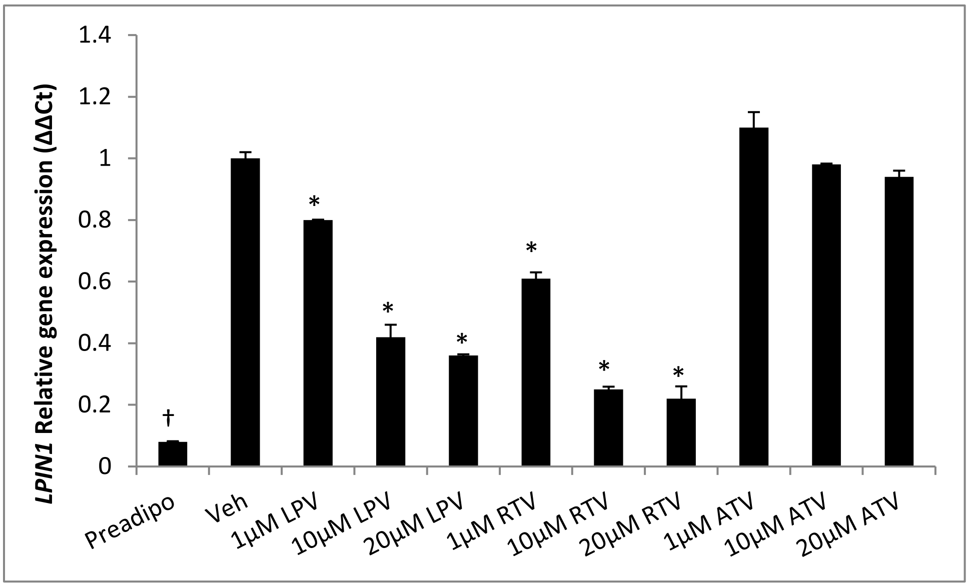
LPIN1 Relative gene expression (ΔΔCt)
1.4
1.2
1
0.8
0.6
0.4
0.2
0
†
*
*
*
*
*
*
Preadipo
Veh
1µM LPV
10µM LPV
20µM LPV
1µM RTV
10µM RTV
20µM RTV
1µM ATV
10µM ATV
20µM ATV

C)

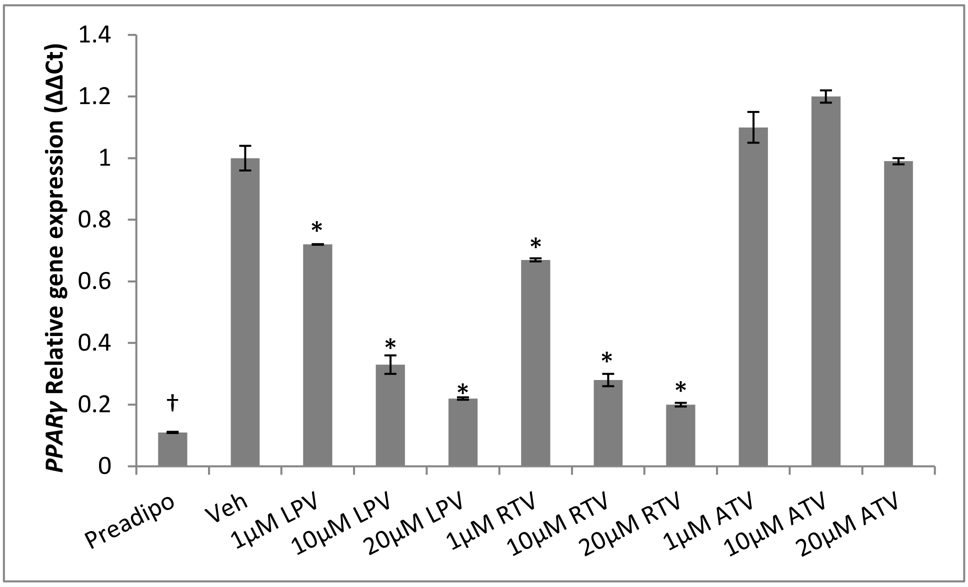
PPARγ Relative gene expression (ΔΔCt)
1.4
1.2
1
0.8
0.6
0.4
0.2
0
†
*
*
*
*
*
*
Preadipo
Veh
1µM LPV
10µM LPV
20µM LPV
1µM RTV
10µM RTV
20µM RTV
1µM ATV
10µM ATV
20µM ATV

D)

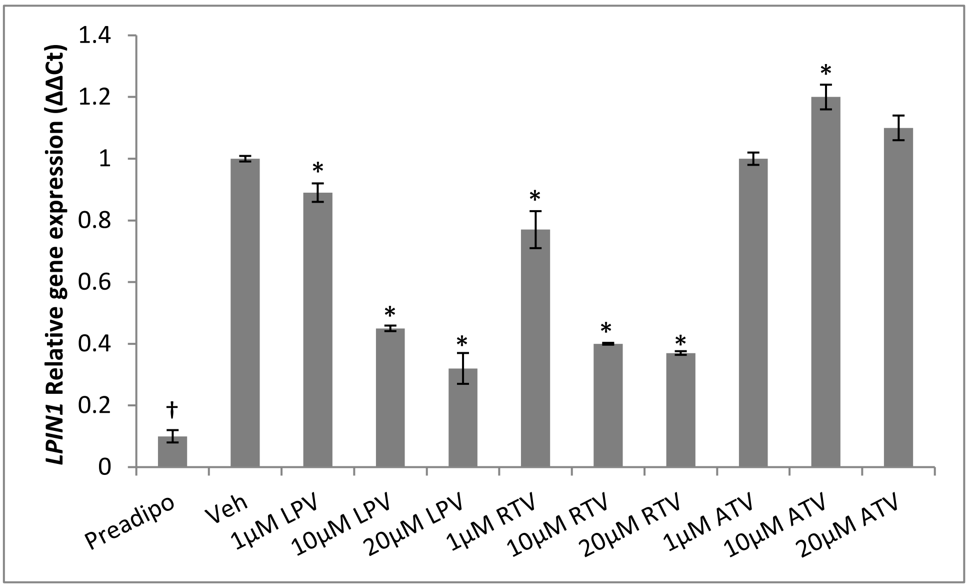

Figure 2: Adipogenic gene expression in differentiating 3T3-F442A (A&B) and human primary adipocytes (C&D) following incubation with serial concentrations of protease inhibitors.

Data expressed as mean (n=3) ± SD.
*=P<0.01; Drug v Vehicle. †=P<0.01; Preadipocyte v Vehicle.
Preadipo: preadipocyte; Veh: vehicle; LPV: lopinavir; RTV: ritonavir; ATV: atazanavir.

A)

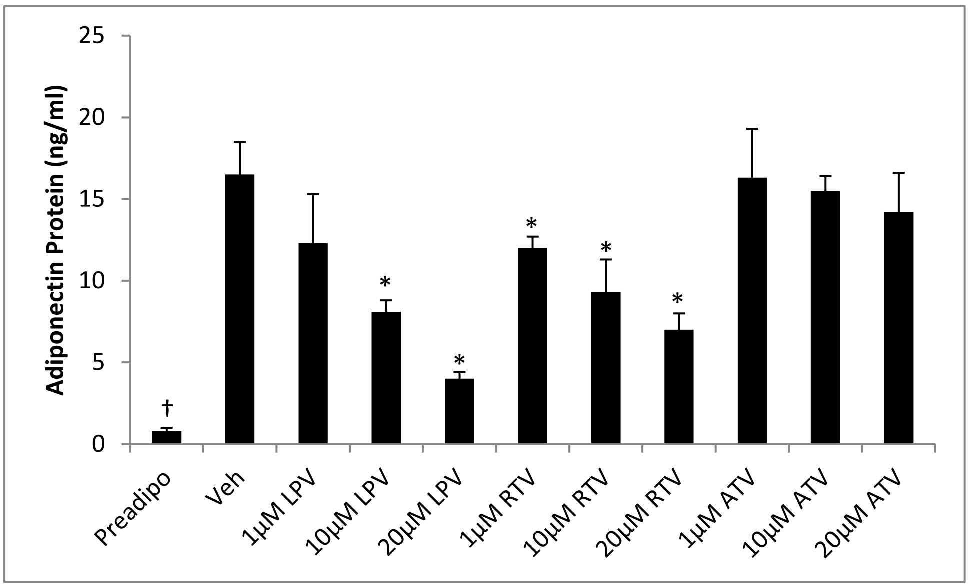

B)

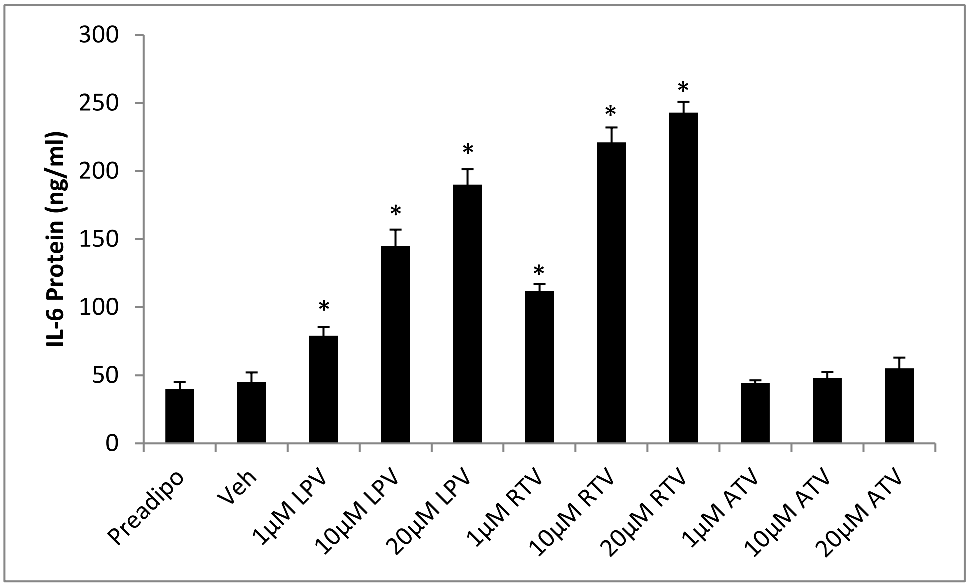
IL-6 Protein (ng/ml)
300
250
200
150
100
50
0
*
Preadipo
Veh
1µM LPV
10µM LPV
20µM LPV
1µM RTV
10µM RTV
20µM RTV
1µM ATV
10µM ATV
20µM ATV

C)

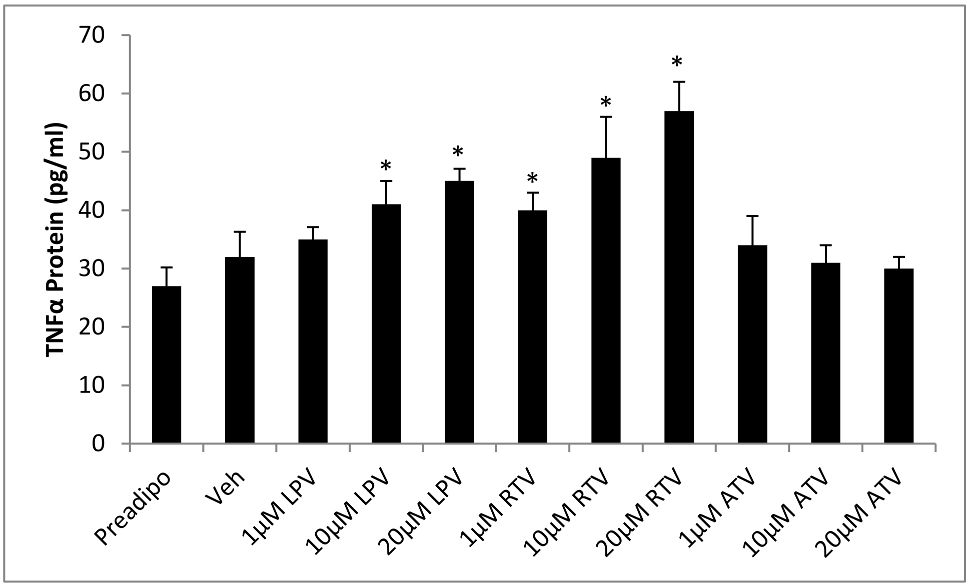
TNFα Protein (pg/ml)
70
60
50
40
30
20
10
0
*
Preadipo
Veh
1µM LPV
10µM LPV
20µM LPV
1µM RTV
10µM RTV
20µM RTV
1µM ATV
10µM ATV
20µM ATV

D)

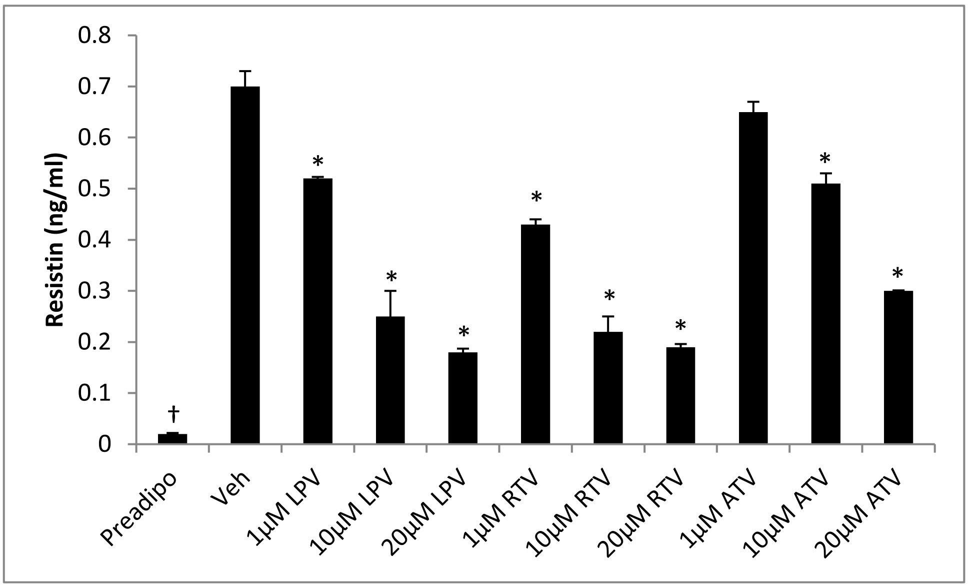
Resistin (ng/ml)
0.8
0.7
0.6
0.5
0.4
0.3
0.2
0.1
0
†
*
Preadipo
Veh
1µM LPV
10µM LPV
20µM LPV
1µM RTV
10µM RTV
20µM RTV
1µM ATV
10µM ATV
20µM ATV

E)

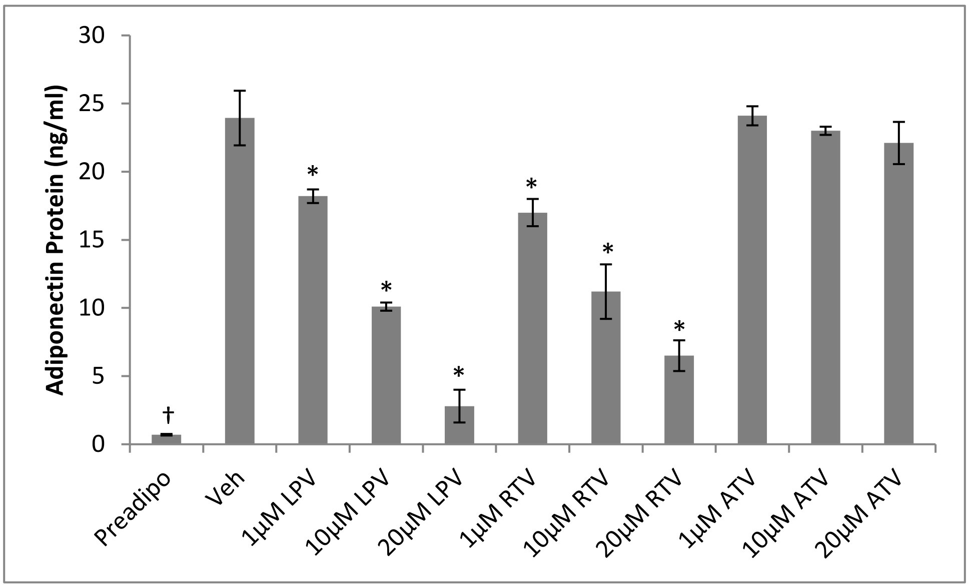

Adiponectin Protein (ng/ml)
30
25
20
15
10
5
0
†
*
Preadipo
Veh
1µM LPV
10µM LPV
20µM LPV
1µM RTV
10µM RTV
20µM RTV
1µM ATV
10µM ATV
20µM ATV

F)

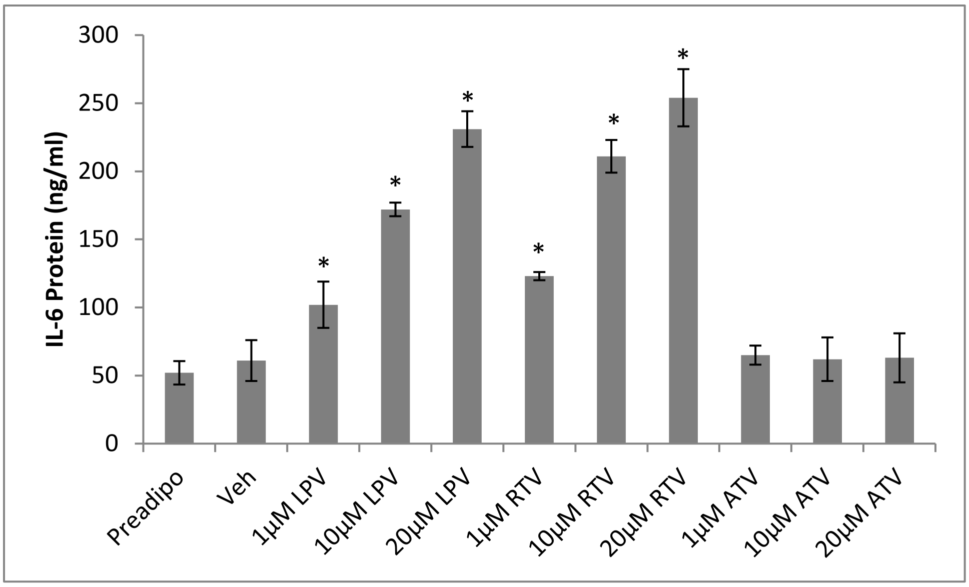

IL-6 Protein (ng/ml)
300
250
200
150
100
50
0
*
Preadipo
Veh
1µM LPV
10µM LPV
20µM LPV
1µM RTV
10µM RTV
20µM RTV
1µM ATV
10µM ATV
20µM ATV

G)

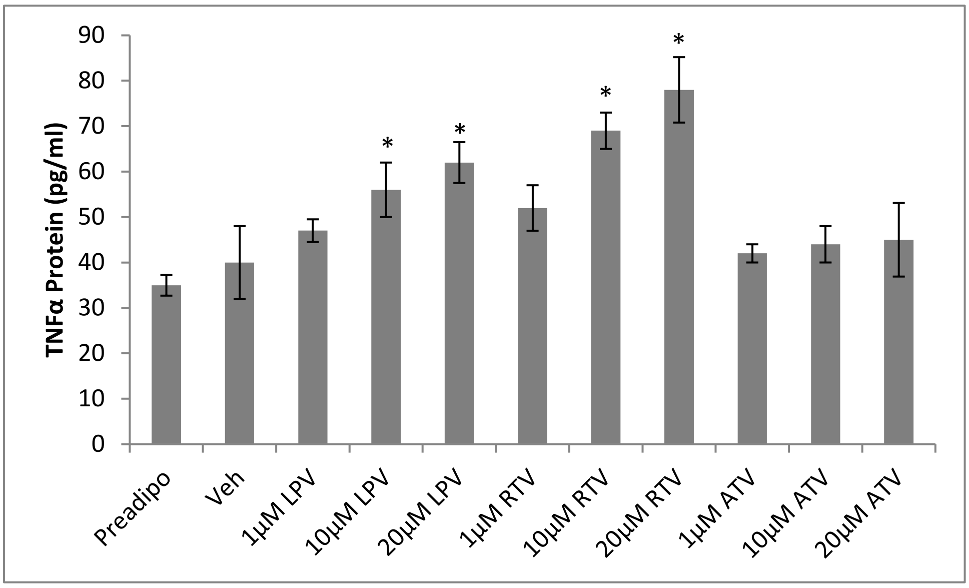

TNFα Protein (pg/ml)
90
80
70
60
50
40
30
20
10
0
*
Preadipo
Veh
1µM LPV
10µM LPV
20µM LPV
1µM RTV
10µM RTV
20µM RTV
1µM ATV
10µM ATV
20µM ATV

H)

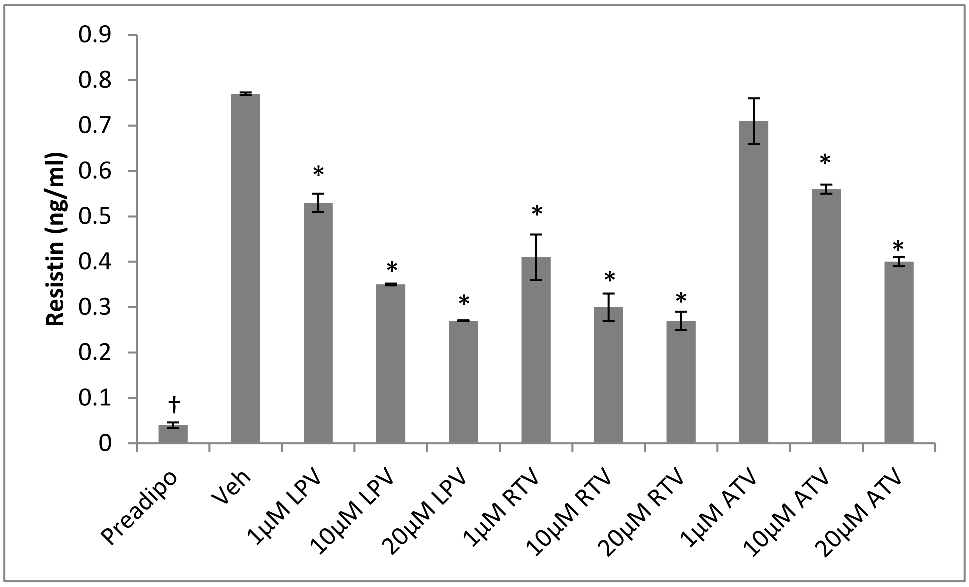

Figure 3. Adipokine secretion in differentiating 3T3-F442A (A – D) and primary human adipocytes (E–H) following incubation with serial concentrations of protease inhibitors.

3T3-F442A adipocytes: A) Adiponectin; B) IL-6; C) TNF-α and; D) Resistin.
Human primary adipocytes: E) Adiponectin; F) IL-6; G) TNF-α and; H) Resistin.
[a]Data expressed as mean (n=3) ± SD. *=P<0.01; Drug v Vehicle. †=P<0.01; Preadipocyte v Vehicle. Preadipo: preadipocyte; Veh: Vehicle; RTV: ritonavir; LPV: lopinavir; ATV: atazanavir; IL-6: Interleukin-6. TNFα: Tumor necrosis factor-α.

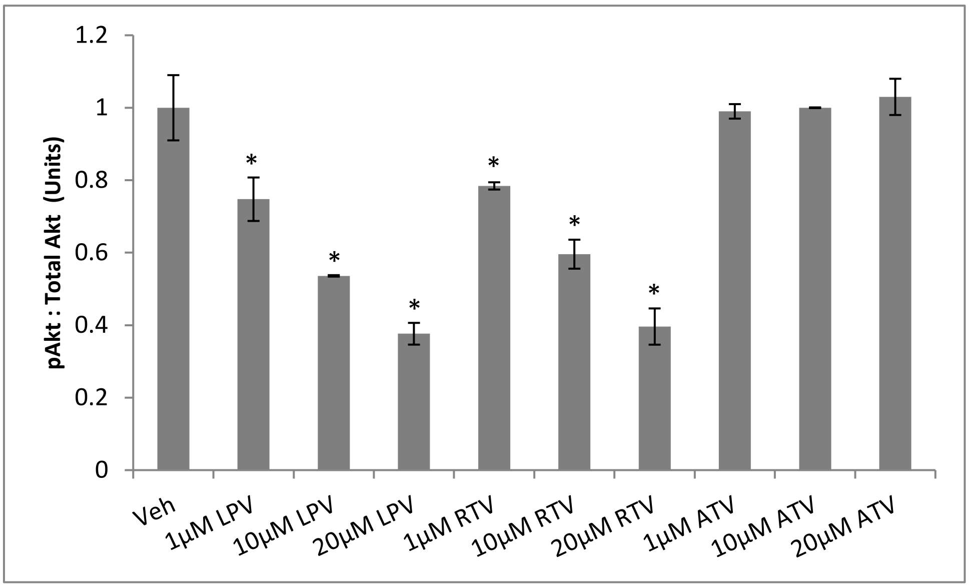

Figure 4. Expression of $pAkt_{Ser473}$ in differentiating primary human adipocytes following incubation with serial concentrations of protease inhibitors.

Data expressed as mean ratio of absorbance (pAkt expression adjusted to Total Akt).
Data expressed as mean (n=3) ± SD.
*=P<0.01; Drug v Vehicle. Veh: Vehicle; RTV: ritonavir; LPV: lopinavir; ATV: atazanavir.
